# A machine learning approach for predicting descending thoracic aortic diameter

**DOI:** 10.3389/fcvm.2023.1097116

**Published:** 2023-02-13

**Authors:** Ronghuang Yu, Min Jin, Yaohui Wang, Xiujuan Cai, Keyin Zhang, Jian Shi, Zeyi Zhou, Fudong Fan, Jun Pan, Qing Zhou, Xinlong Tang, Dongjin Wang

**Affiliations:** ^1^Medical School, Department of Cardio-Thoracic Surgery, Affiliated Drum Tower Hospital, Nanjing University, Nanjing, China; ^2^Nanjing Drum Tower Hospital, Clinical College of Nanjing Medical University, Nanjing, China; ^3^Shanghai Artificial Intelligence Laboratory, Shanghai, China; ^4^Nanjing Drum Tower Hospital, Clinical College of Nanjing University of Chinese Medicine, Nanjing, China

**Keywords:** machine learning, aortic diameter, TEVAR, CTA, predictive model

## Abstract

**Background:**

To establish models for predicting descending thoracic aortic diameters and provide evidence for selecting the size of the stent graft for TBAD patients.

**Methods:**

A total of 200 candidates without severe deformation of aorta were included. CTA information was collected and 3D reconstructed. In the reconstructed CTA, a total of 12 cross-sections of peripheral vessels were made perpendicular to the axis of flow of the aorta. Parameters of the cross sections and basic clinical characteristics were used for prediction. The data was randomly split into the training set and the test set in an 8:2 ratio. To fully describe diameters of descending thoracic aorta, three predicted points were set based quadrisection, and a total of 12 models at three predicted points were established using four algorithms included linear regression (LR), support vector machine (SVM), Extra-Tree regression (ETR) and random forest regression (RFR). The performance of models was evaluated by mean square error (MSE) of the prediction value, and the ranking of feature importance was given by Shapley value. After modeling, prognosis of five TEVAR cases and stent oversizing were compared.

**Results:**

We identified a series of parameters which affect the diameter of descending thoracic aorta, including age, hypertension, the area of proximal edge of superior mesenteric artery, etc. Among four predictive models, all the MSEs of SVM models at three different predicted position were less than 2 mm^2^, with approximately 90% predicted diameters error less than 2 mm in the test sets. In patients with dSINE, stent oversizing was about 3 mm, while only 1 mm in patients without complications.

**Conclusion:**

The predictive models established by machine learning revealed the relationship between basic characteristics and diameters of different segment of descending aorta, which help to provide evidence for selecting the matching distal size of the stent for TBAD patients, thereby reducing the incidence of TEVAR complications.

## Introduction

Type B aortic dissection (TBAD) is a life-threatening aortic disease. The thoracic endovascular aortic repair (TEVAR) procedure, involving the placement of a covered stent into the weakened area of the artery, has been recommended as a standard treatment for TBAD, especially for some complicated conditions, such as rapid growing of the diameter of the dissected aorta and impending rupture signs (1). However, complications after TEVAR, such as the endoleak and distal stent graft-induced new entry (dSINE), are substantial concerns (2), and reoperation is even required.

The mismatch of the stent size and the descending thoracic aorta has been considered as a significant cause of TEVAR complications (1, 3–5). Because of the compressed true lumen and the expanded adventitia caused by the false lumen, it’s difficult to choose stents matching the true diameter of the thoracic descending aorta based on the measured CT results (6, 7). Lettinga-van et al. reported an approximately 6.7% endoleak rate after TEVAR caused by an insufficient (proximal/distal) seal (8). To achieve a sufficient seal, the size of stent has been generally guided by 10∼20% larger than the measured diameter of the proximal landing zone of aorta (1, 9, 10), without more requirements on the diameter of distal aorta. However, due to the increased aortic taper ratio, stent can be too large for the distal edge to avoid dSINE (3, 4). Therefore, it is of great significance to establish a predictive model for the diameter of descending thoracic aorta and guide the selection of matching stent size to reduce the complications of TEVAR.

In recent years, some researchers have explored the impact of age, gender, peripheral vascular conditions, etc., on the diameter of the descending aorta (11–13). However, due to the small number of variables included or the poor fitting effect of models, there are few models for predicting the diameter of the descending aorta so far. Based on the assumption that the descending thoracic aortic dissection can occur in the absence of significant aortic dilation (14, 15), we collected CTA from 200 candidates without diseases which cause severe deformation of aorta, and employed machine learning to predict the diameter of descending aorta in normal morphology. It is expected that our machine learning models can reveal the relationship between the basic physical condition and the descending aortic diameter under normal hemodynamic conditions, and provide the evidence for selecting the matching distal size of the stent for TBAD patients without aneurysm, thereby reducing the incidence of TEVAR complications.

## Materials and methods

### Dataset

We selected patients who underwent CTA examination in Nanjing Drum Tower Hospital from 2019 to 2022. All aortic measurements were read in a standardized manner and confirmed by two senior investigators (JM and TL). During modeling, considering that aortic morphology can be seriously affected by aortic diseases such as aneurysm, consulting official radiology reports, the exclusion criteria included: any types of aortic dissection, aneurysm, aortic tortuosity, stent graft implantation and vascular replacement. Among aortic diseases, aortic dilatation was considered morphologically continuous and predictable, unlike aortic aneurysms, which have sudden aortic morphological changes, and was not excluded. Finally, our dataset for modeling consisted of 200 samples and each sample contains 88 features to predict the diameter of the descending aorta. Seventy-two features were obtained from measurement of arteries, and the measuring method was described in the following “measurement and models” section. The 16 basic patient characteristics included: gender, age, height(m), weight(kg), Body Mass Index (BMI) (kg/m^2^), hypertension, blood pressure control, dyslipidemia, diabetes, smoke, alcohol, autoimmune disease (AD), myocardial infarction (MI), stable/unstable angina, chronic kidney disease (CKD) and stroke. The AD involved any type of disease related to autoimmune disorder, and in our datasets, there were five Hashimoto’s thyroiditis, four arteritis, one Sjogren Syndrome, one leukoderma, one psoriasis and one other connective tissue diseases. After modeling, We reported five TEVAR cases and studied the effect of stent size on prognosis.

### Measurement and models

**FIGURE 1:**
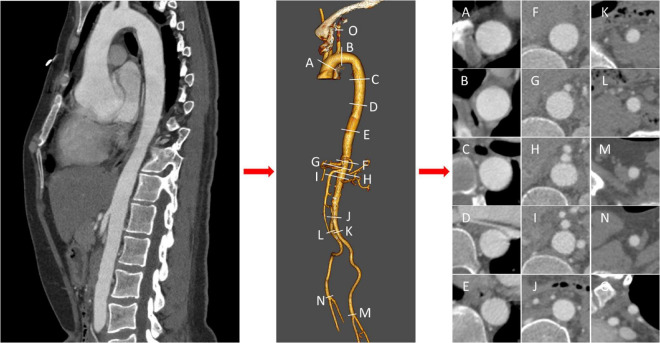
Schematic diagram of measurements. After three-dimensional reconstruction of thoracic and abdominal aorta, landmarks were measured orthogonal to the centerline at labeled position **(A–O)**, and cross sections were acquired.

An experienced radiologist unaware of the purpose of the study was responsible for the CT measurement data acquisition. Endosize 3.1.40 was used for 3D reconstruction and measurement of the aorta of patients. Briefly, after the candidates’ CTA was imported into Endosize, the central line was determined with the aortic sinus as the start point and the left/right profound femoral arteries as the end points. At the same time, the central line of the left subclavian artery branch was established for 3D reconstruction of the aorta. According to the landmarks recommended in the 2014 ESC Guidelines on the diagnosis and treatment of aortic diseases with some modifications, a total of 15 measurement points were determined: aorta at the proximal edge of innominate artery (A), aorta at the distal edge of left subclavian artery (B), aorta at the proximal edge of celiac trunk (F), aorta at the proximal edge of superior mesenteric artery (G), aorta at the proximal edge of the left renal artery (H), aorta at the proximal edge of the right renal artery (I), distal edge of the abnormal artery (J), distal edge of left iliac artery (K), distal edge of right iliac artery (L), distal edge of left femoral artery (M), distal edge of right femoral artery (N), left subclavian artery at the proximal edge of vertebral artery (O). C, D, and E were defined as the 1/4, 1/2 and 3/4 points of B and F, respectively. Figure 1 showed the schematic diagram of measuring. During the measurement, cross-sections were made perpendicular to the axis of flow of the aorta, consistent with the previous studies (16). In the Endosize advanced measurement mode, a total of six measurements were obtained from each cross-section: cross-section perimeter (Peri), cross-section Area (Area), maximum diameter of cross-section (Max), minimum diameter of cross-section (Min), perimeter-derived diameter (diameterP), area-derived diameter (diameterA). The diameterP of C, D and E were defined as the diameter of the descending aorta and denoted as y_1_, y_2_, y_3_ for prediction. Thus, a total of 72 (6 × 12) geometric features were acquired as input features.

In our experiments, we split the whole dataset into train and test sets with the split rate of 8:2. Four machine learning methods were applied to predict the value of y_1_, y_2_, y_3_, i.e., linear regression (LR) (17), random forest regression (RFR) (18), support vector machine (SVM) (19) and Extra-Trees regression (ETR) (20) on our dataset. We introduce four methods in detail in the following sections. Shapley Additive Explanations (SHAP) were used to define feature importance.

### LR

LR fits a linear model with coefficients W = (w_1_, w_2_,…, w_*N*_) by minimizing the residual sum of squares between y_*i*_ in the dataset and the f_*i*_(x) by the linear approximation.

### RFR

RFR fits several classifying decision trees on various sub-samples of the datasets selected by bootstrap method. After selecting the splitting feature, an optimal eigenvalue was determined as the splitting point based on information divergence, Gini coefficient and mean square error, etc. It uses averaging to improve the predictive accuracy and control over-fitting. We set the maximum depth of each tree as 2 in our experiments.

### SVM

We used support vector regression (SVR) to solve the regression problem by constructing the hyperplane with the shortest distance from each sample point. In our experiment, we choose to use linear kernel as our sample size is relatively small.

### ETR

ETR fits some randomized decision trees (a.k.a. Extra-Trees) on the original training set. The features are always randomly permuted at each split, and splitting points are selected randomly. It also uses averaging to improve the predictive accuracy and control over-fitting. In our experiments, we used 20 estimators.

### SHAP

The Shapley value accounts for the marginal contribution of features. For each feature of a single sample, the shapley value is calculated according to its marginal contribution, and the final prediction is the sum of the base value and the marginal contributions of all the features.

### Problem formulation

Our objective is to learn a set of mapping functions f ∈ {f_1_, f_2_, f_3_}, to map each sample x ⊂χ_*N*_ into corresponding y_1_, y_2_, y_3_ ⊂ Y_*M*_, such as f_*i*_(x) = y_*i*_, i = 1, 2, 3, where N represents 72 features, M represents 3 predicted points.

### Statistical analysis

All data are presented as n (%) for categorical variables and mean with standard deviation for normally distributed continuous variables. Normality distribution were tested with the Kolmogorov-Smirnov test. Independent *t*-tests were performed for normally distributed variables, or Mann-Whitney U tests for non-normal distribution. Categorical variables were analyzed by Chi-square test or Fisher’s exact test. R software (version 4.0.3) was used for data analysis. R packages “tableone” were used for basic statistics and to make table one. Python (version 3.10) was used to build the models using packages “LinearRegression,” “svm,” “ExtraTreesRegressor,” and “RandomForestRegressor” of sklearn. MSE was calculated as: MSE = $\frac{1}{n}\times \sum_{j=1}^{n}{\left(p\,r\,e\,d\,i\,c\,t\,e\,d\,y_{i\,j}-y_{i\,j}\right)}^{2}$. A p-value of less than 0.05 was considered statistically significant.

## Results

### Cohort description

Table 1 shows the basic characteristics of candidates. Among them, 63.5% were males with a mean age of 62. The median height is 168 cm with the first quartile 173 cm and the third quartile 160 cm. The median body weight was 65.5 kg with the first quartile 59 kg and the third quartile 72.62 kg. Unsurprisingly, many candidates had significant known comorbidities for aortic disease, including a large percentage with hypertension (44.5%), dyslipidemia (22.0%), stable/unstable angina (17.5%) and myocardial infarction (3.5%). “Satisfied blood pressure control” was considered as someone who has a history of hypertension, but the blood pressure is less than 130/80 mmHg under standard measuring method after lifestyle improvement or drug control. In the population with hypertension disease, 73.3% of candidates with hypotensive drugs such as beta blockers or calcium channel blockers achieved satisfied blood pressure control. According to the aortic dilation diagnostic criteria of greater than 40 mm, there were 23 candidates with aortic dilatation. The training set and test set were split by a ratio of 8:2, which contained 160 training samples and 40 testing samples. *P*-values of all the features were > 0.05 between training and testing sets (Supplementary Table 2), showing no significant difference. None of the samples had missing values.

**TABLE 1:** Basic characteristics of candidates.

Basic characteristic		Value
Sample number		200
Gender	Male, *n* (%)	127(63.5)
	Female, *n* (%)	73 (36.5)
Age (median [IQR])		62.00 [47.00, 71.00]
Height (median [IQR])		168.00 [160.00, 173.00]
Weight (median [IQR])		65.50 [59.00, 72.62]
BMI (median [IQR])		23.85 [21.19, 25.78]
Hypertension, *n* (%)		89 (44.5)
Blood pressure control, *n* (%)		60 (30.0)
Dyslipidemia, *n* (%)		44 (22.0)
Diabetes, *n* (%)		24 (12.0)
Smoke, *n* (%)		30 (15.0)
Alcohol, *n* (%)		23 (11.5)
Autoimmune Disorder, *n* (%)		14 (7.0)
MI, *n* (%)		7 (3.5)
Angina, *n* (%)		35 (17.5)
CKD, *n* (%)		1 (0.5)
Stroke, *n* (%)		7 (3.5)

Age, height, weight and BMI were showed as mean with IQR. IQR, interquartile range. MI, myocardial infarction. CKD, chronic kidney disease.

### Models performance

**FIGURE 2:**
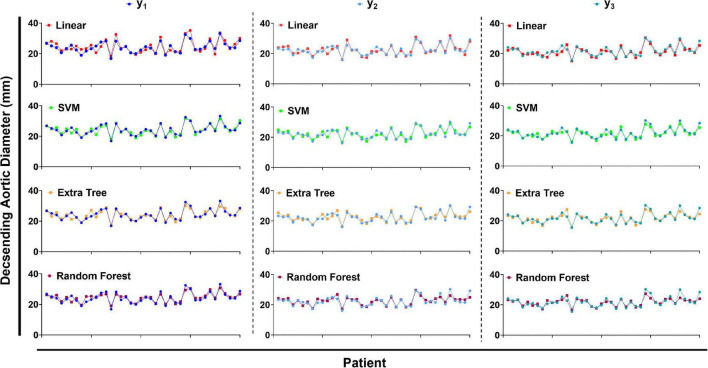
The predicted values of descending aortic diameter compared with the true values. LR, linear regression. SVM, support vector regression. ETR, extra-tree regression. RFR, random forest regression.

**FIGURE 3:**
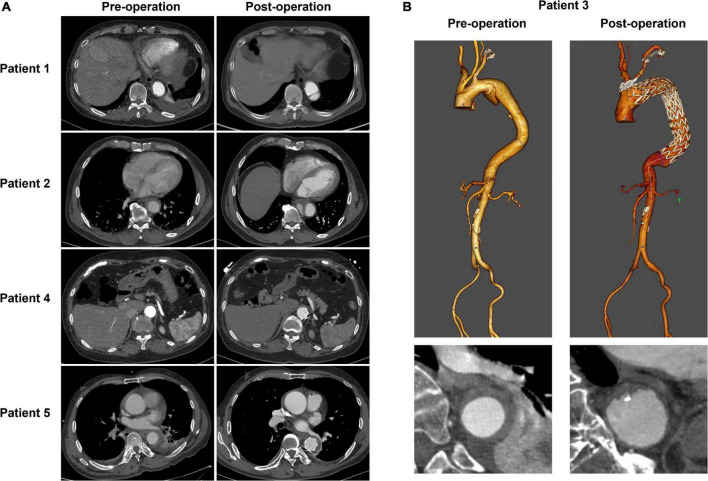
The CTA image changes of five TEVAR patients during follow-up. **(A)** The cross-section images of patient1, 2, 4, and 5. **(B)** 3D reconstruction of CTA image of patient 3.

Table 2 summarizes the performance of a total of 12 models, using four machine learning predictive models at three descending aorta positions of prediction. The LR gave the largest MSE in the test sets, perhaps due to a lack of linear relationship between descending aortic diameter and included parameters. The SVM model performed the best with the lowest MSE, although it constructed a most complicated model accompanied by the largest number of important features. All the SVM MSEs were less than 2 mm^2^, and the corresponding error between the predicted diameters and the real values is less than 2 mm in approximately 90% samples, showing the powerful role of machine learning in the study of aortic morphology. Figure 2 compares the predicted values of various models in the test set and the relative real values at C, D and E points. Obviously, the predicted values of the 4 models are very close to the actual values, demonstrating the decisive role of machine learning in predicting the diameter of the descending aorta. Table 3 compares the prognosis of five TEVAR patients, and basic characteristics of patients are listed in Supplementary Table 3. We used the SVM model to predict the diameter of descending thoracic aorta on five patients. According to the position of distal edge of stent graft in descending thoracic aorta, corresponding descending aortic diameters were calculated using CTA image before TEVAR operation. The stent oversizing levels (OL) were estimated as: OL = stent diameter – SVM predicted diameter. Stent oversizing level of patients were about 3 mm, 3mm, 4 mm, 1 mm, 1 mm, respectively. Patient 1, 2 and 3 suffered dSINE 4/1/10 month(s) after the first TEVAR, respectively, and reoperation was required. In contrast, patient 4 and 5 did not develop any TEVAR complication after 10/11 months follow-up, respectively. Figure 3 shows the CTA image changes during follow-up.

**TABLE 2:** Performance of models in test and train sets.

Models		Train error	MSE	Numbers of selected features
LR	Diameter of C	1.41	4.81	30
	Diameter of D	0.84	2.54	28
	Diameter of E	0.73	2.52	22
SVM	Diameter of C	2.68	1.86	34
	Diameter of D	1.41	1.82	37
	Diameter of E	1.48	1.91	38
ETR	Diameter of C	3.28 × 10^–29^	2.43	25
	Diameter of D	3.24 × 10^–29^	2.04	21
	Diameter of E	2.49 × 10^–29^	2.34	17
RFR	Diameter of C	3.85	2.51	20
	Diameter of D	2.30	2.73	17
	Diameter of E	2.08	3.00	15

LR, linear regression. SVM, support vector regression. ETR, extra-tree regression. RFR, random forest regression.

**TABLE 3:** Validation of TEVAR patients.

	Position X	Predicted diameter of X (mm)	Stent size (mm)	Stent oversizing (mm)	Complications
Patient 1	D	27.13	30	+ 2.88	dSINE
Patient 2	D	25.15	28	+ 2.85	dSINE
Patient 3	E	23.75	28	+ 4.25	dSINE
Patient 4	E	25.00	26	+ 1.00	None
Patient 5	D	25.62	26	+ 0.40	None

Position X, the position of distal stent landing zone at descending aorta. Definitions of D/E are illustrated in Figure 1. Predicted diameter of X, the Predicted diameter of X using SVM model. Stent size, TEVAR stent size at position X.

### Feature importance

**FIGURE 4:**
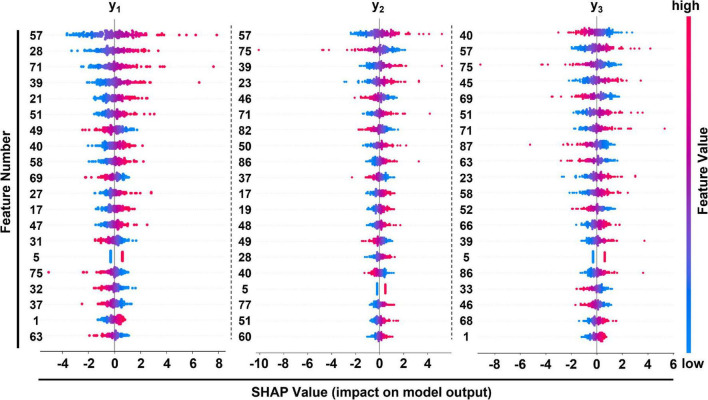
Summary plot of feature importance in SVM model at 3 different positions of prediction. Corresponding features of feature numbers were listed in Supplementary Table 1.

**FIGURE 5:**
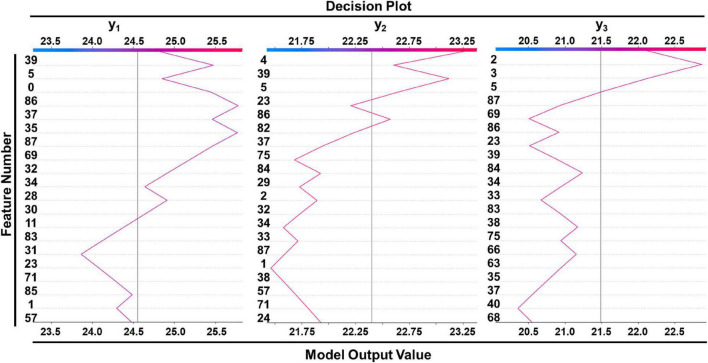
Decision plot of a single sample showed how included features affected prediction in SVM model. Corresponding features of feature numbers were shown in Supplementary Table 1.

Table 4 lists the selected critical features in different models based on importance weights. In the test set, SVM selected the largest number of important features, with more than 30 features in each of the three prediction sites. Random Forest selected the least number of important features. ETR-Tree had relatively few features and a small MSE. The LR has more selected features, but the MSE was also the largest. We further studied the contribution of top 20 features in the SVM model using the Shapley value (Figure 4). In the summary plot, each point represented the corresponding feature value and shapley value of one single sample, and the corresponding feature names of numbers are shown in Supplementary Table 1. Shapley values demonstrated a positive relationship between age, hypertension and descending aortic diameter, as a higher feature value is associated with a greater marginal contribution and a higher shapley value. A weak marginal contribution of height, weight and BMI was observed. No surprisingly, Blood pressure control presented a negative effect for the prediction. For fundamental disease only hypertension was included in important features of models, indicating minor effects of others. Since the SVM model was the most accurate and more than 30 important features were selected, we further explored the top 40 important features in SVM models in Supplementary Figures 1–3. The decision plot (Figure 5) shows how the marginal contribution of each feature affected the predicted diameters in one single sample using SVM model. For the prediction of y_1_, the characteristics “female” (feature 0) and “hypertension” (feature 5) leading to a marginal contribution of minus 0.60 mm and plus 0.63 mm in the presented sample, respectively. The predicted value of y_1_ “24.55 mm” was the sum of all the marginal contributions of features.

**TABLE 4:** Selected features of different models.

Models		Selected features
LR	Diameter of C	16 18 19 20 30 31 36 37 38 39 43 48 49 50 56 57 61 62 66 67 68 71 73 74 78 80 81 84 85 86
	Diameter of D	18 19 20 24 25 31 36 37 38 42 44 45 48 49 50 54 55 56 57 60 62 67 68 73 75 84 85 86
	Diameter of E	18 19 20 36 37 38 42 44 45 48 50 55 56 60 61 62 67 68 72 74 84 85
SVM	Diameter of C	0 1 5 13 17 21 22 23 27 28 30 31 32 34 35 37 39 40 42 45 47 49 51 57 58 63 69 71 75 76 77 79 86 87
	Diameter of D	1 3 4 5 13 17 19 21 23 24 28 29 32 37 39 40 41 43 44 45 46 48 49 50 51 52 57 60 63 66 70 71 75 77 82 84 86
	Diameter of E	1 2 3 4 5 23 24 33 34 35 37 38 39 40 41 43 45 46 47 48 50 51 52 57 58 62 63 64 65 66 68 69 70 71 75 84 86 87
ETR	Diameter of C	1 2 3 4 5 23 24 33 34 35 37 38 39 40 41 43 45 46 47 48 50 51 52 57 58 62 63 64 65 66 68 69 70 71 75 84 86 87
	Diameter of D	53 55 49 54 51 61 48 50 16 46 67 52 17 20 62 5 19 60 56 18 57
	Diameter of E	51 53 54 60 61 49 62 59 50 55 17 48 46 47 63 44 5
RFR	Diameter of C	51 50 17 53 47 16 21 54 56 59 55 58 49 19 48 57 60 44 18 20
	Diameter of D	50 53 51 47 48 57 60 62 41 16 55 49 56 45 17 18 52
	Diameter of E	47 50 51 53 62 60 48 16 56 57 61 17 49 55 45

Selected features of 4 different machine learning models at 3 predicted positions. Corresponding features of feature numbers were shown in Supplementary Table 1.

## Discussion

Machine learning has been increasingly applied in medical research in recent decades, making a significant impact on the practice of medicine, from image recognition to GWAS studies of genetic loci that influence the diameter of the aorta (21). To our knowledge, this is the first research using machine learning to predict descending thoracic aortic diameters, and all four developed models performed well (maximum MSE = 1.91 mm^2^, maximum standard estimated error (SEE) = 1.38 mm in SVM model). Compared with the previous studies, our models achieved satisfying prediction accuracy (minimum SEE = 2.15 mm) (11, 13, 22).

Of note, all models included parameters of different segments of arteries, and a positive contribution to the predicted value was acquired accompanied by a higher feature value, which is also consistent with our impression of measurement: arterial widening tends to be systemic; a wider descending aorta is often accompanied by wider peripheral arteries such as the femoral artery. Shapley values show that the area of proximal edge of superior mesenteric artery is positively correlated with all three predicted diameters as one of the top important features. We speculated that it’s because of anatomical proximity. There are also paradoxical results, for example, the maximum diameter of the terminal abdominal aorta was positively correlated with the diameter of the descending aorta, while the cross-sectional area of the terminal abdominal aorta showed a negative effect in all three predicted positions. We suspected that the maximal diameter of the terminal abdominal aorta represents an anatomical continuation, while the effect of the abdominal aortic terminal cross-sectional area is the result of hemodynamics, as severe abdominal aortic constriction can fully induce pressure overload and subsequent heart failure (23–25). Our work can be compared with previous study by Takashi Yamauchi and colleagues, who also investigated in the estimation of descending thoracic aortic diameter (26). By taking an average perimeter of whole lumen and true lumen after aortic dissection as the pre-dissection perimeter, they achieved a bias of less than 2 mm in 90% cases. However, due to the small number of cases in which data before and after aortic dissection could be obtained, they only included a total of 36 cases, among them 17 for modeling and 19 for validation.

Our research leaves much to be desired. We based on the hypothesis that the descending aorta does not appear to have an acute dilatation before aortic dissection, but as a matter of fact, it is difficult to validate the hypothesis as we can barely obtain the data just before aortic dissection happens. In terms of research design, the sample size is still not enough, and the features we take into account are not comprehensive, such as NT-ProBNP, C-reaction protein, hereditary background, central pulse pressure (22, 27–29), etc. In addition, the model we established requires too many parameters to be used conveniently in clinical practice. Finally, whether it can be applied in deciding stent graft size in TEVAR needs further validation in clinical situations.

In general, our study provides the first predictive models using machine learning to reveal the relationship between descending aortic diameter and basic human characteristics under normal hemodynamic conditions, which is expected to provide evidence for the selection of stent graft size in patients with type B aortic dissection, thereby decreasing TEVAR complication incidence and reducing healthcare burden.

## Conclusion

Descending thoracic aortic diameter has a complicated relationship with basic characteristics such as age, gender, hypertension and peripheral blood vessels, etc. Machine learning can accurately predict the diameter of descending thoracic aorta based on these features and reveal the morphological rules of descending aorta.

## Data availability statement

The original contributions presented in this study are included in this article/Supplementary material, further inquiries can be directed to the corresponding authors.

## Ethics statement

The studies involving human participants were reviewed and approved by Nanjing Drum Tower Hospital (2020-185-01). The ethics committee waived the requirement of written informed consent for participation. Written informed consent was not obtained from the individual(s) for the publication of any potentially identifiable images or data included in this article.

## Author contributions

RY was responsible for analyzing the data, drafting the initial manuscript, and critically revising the manuscript. XC and KZ helped collecting the data. YW was the main contributor to the deep learning algorithm. ZZ and JS helped interpreting the result. MJ and FF revised the manuscript. JP and QZ provided assistance for data acquisition and statistical analysis. XT and DW designed and directed the study, critically reviewed and revised the manuscript. All authors agreed to accept responsibility for this work and agreed on the final manuscript as submitted.
