# Supplementary figures and images for: A machine learning approach for predicting descending thoracic aortic diameter
Source: Front Cardiovasc Med. 2023 Feb 13;10:1097116. doi: 10.3389/fcvm.2023.1097116 (PMC9969122; doi:10.3389/fcvm.2023.1097116)

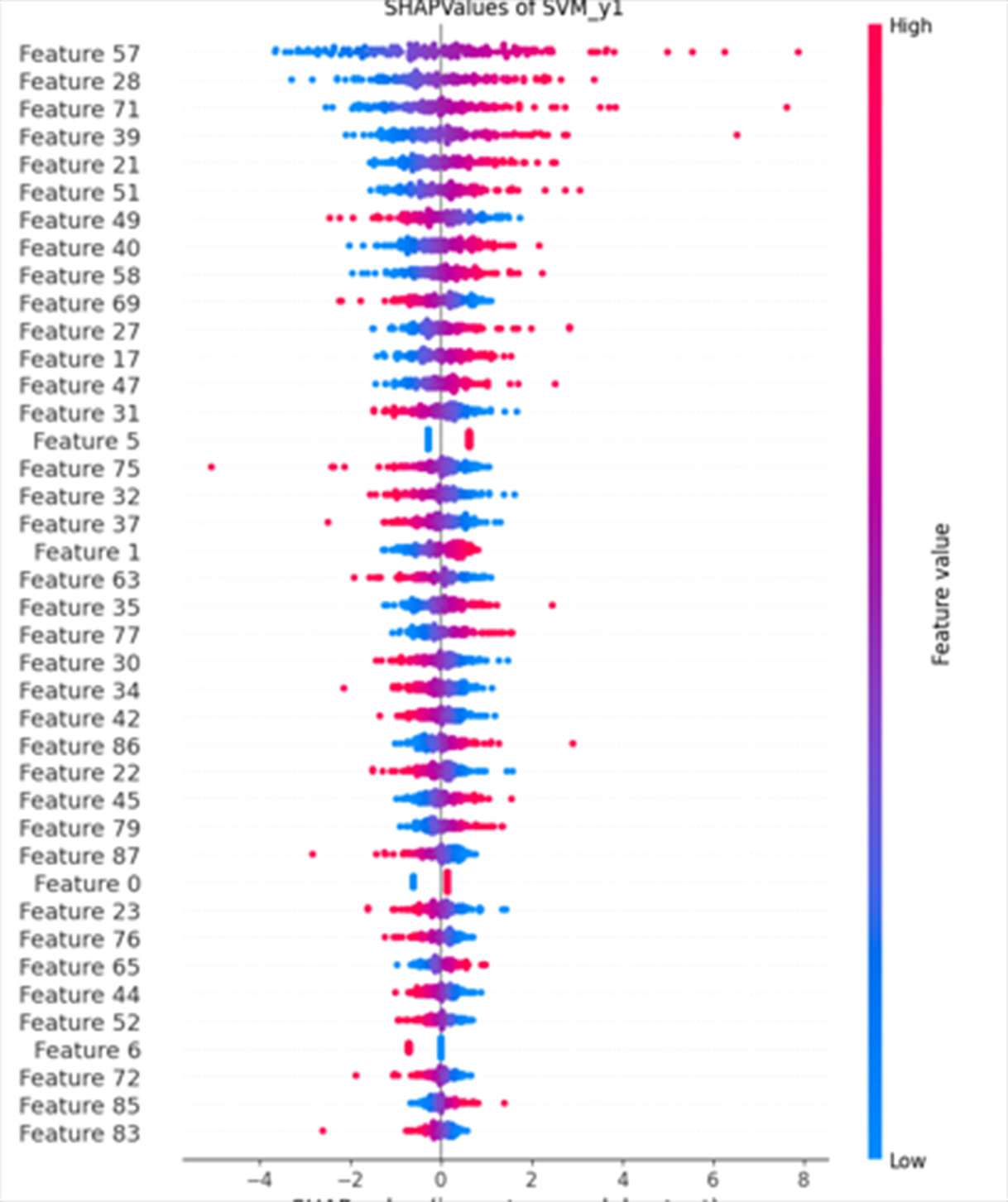

Supplement: Supplementary file 1 [file Image_1.TIF]

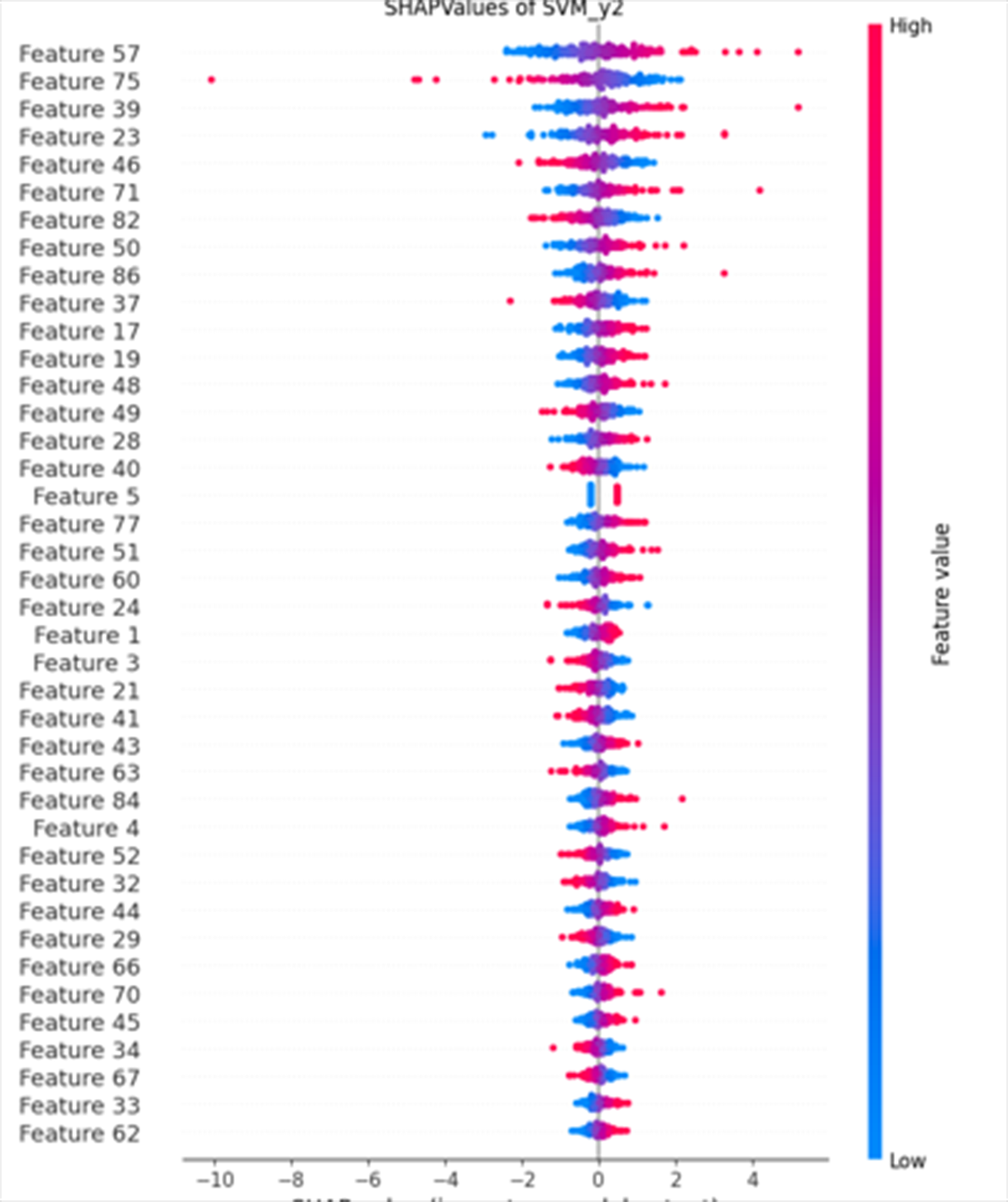

Supplement: Supplementary file 2 [file Image_2.TIF]

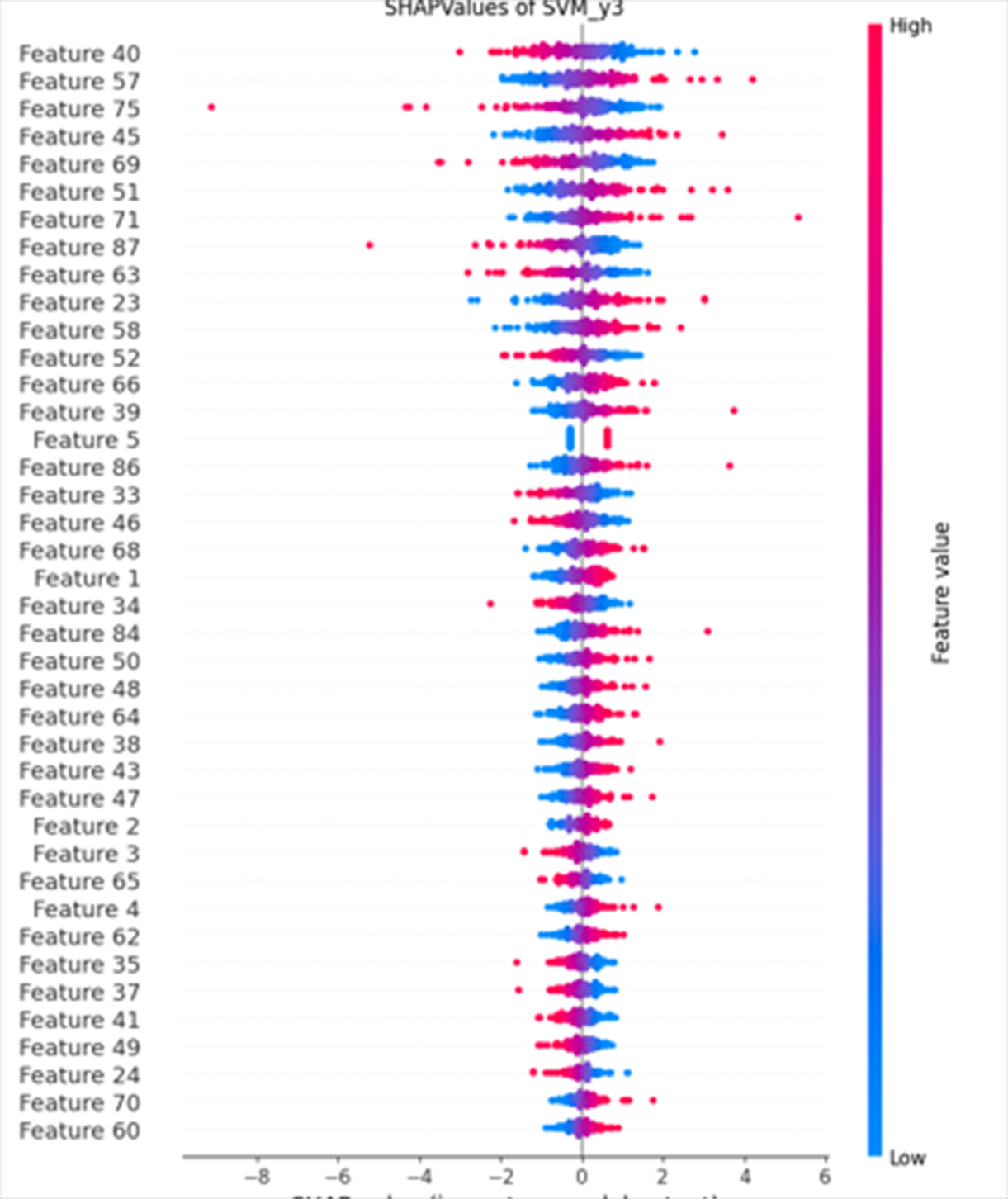

Supplement: Supplementary file 3 [file Image_3.TIF]
