# Supplementary material for: A machine learning approach for predicting descending thoracic aortic diameter
Source: Front Cardiovasc Med. 2023 Feb 13;10:1097116. doi: 10.3389/fcvm.2023.1097116 (PMC9969122; doi:10.3389/fcvm.2023.1097116)
Supplement: Supplementary file 4 [file Table_1.DOCX]

A Machine Learning Approach For Predicting Descending Thoracic Aortic Diameter

**Ronghuang Yu^†^, Min Jin^†^, Keyin Zhang, Jian Shi, Zeyi Zhou, Fudong Fan, Jun Pan, Qing Zhou, Xinlong Tang*, Dongjin Wang***

Department of Cardio-Thoracic Surgery, Affiliated Drum Tower Hospital, Medical School, Nanjing University

**Supplementary Table 1.** Feature Nunber Annotation.

| **Feature Number** | **Feature** | | **Abbreviation** |
| --- | --- | --- | --- |
| 0 | Gender | | Gender |
| 1 | Age | | Age |
| 2 | Height | | Height |
| 3 | Weight | | Weight |
| 4 | Body Mass Index | | BMI |
| 5 | Hypertension | | Hypertension |
| 6 | Blood Pressure Control | | Blood Pressure Control |
| 7 | Dyslipidemia | | Dyslipidemia |
| 8 | Diabetes | | Diabetes |
| 9 | Smoke | | Smoke |
| 10 | Alcohol | | Alcohol |
| 11 | Autoimmune disorder | | Autoimmune disorder |
| 12 | Myocardial Infarction | | MI |
| 13 | Stable/Unstable Angina | | Angina |
| 14 | Chronic Kidney Disease | | CKD |
| 15 | Stroke | | Stroke |
| 16 | Proximal Edge of Innominate Artery | Min | A_min |
| 17 |  | Max | A_max |
| 18 |  | Diameter Calculated From Perimeter | A_diameterP |
| 19 |  | Diameter Calculated From Area | A_diameterA |
| 20 |  | Perimeter | A_Perimeter |
| 21 |  | Area | A_Area |
| 22 | Proximal Edge of Vertebral Artery | Min | O_min |
| 23 |  | Max | O_max |
| 24 |  | Diameter Calculated From Perimeter | O_diameterP |
| 25 |  | Diameter Calculated From Area | O_diameterA |
| 26 |  | Perimeter | O_Perimeter |
| 27 |  | Area | O_Area |
| 28 | Distal Edge of Left Femoral Artery | Min | M_min |
| 29 |  | Max | M_max |
| 30 |  | Diameter Calculated From Perimeter | M_diameterP |
| 31 |  | Diameter Calculated From Area | M_diameterA |
| 32 |  | Perimeter | M_Perimeter |
| 33 |  | Area | M_Area |
| 34 | Distal Edge of Right Femoral Artery | Min | N_min |
| 35 |  | Max | N_max |
| 36 |  | Diameter Calculated From Perimeter | N_diameterP |
| 37 |  | Diameter Calculated From Area | N_diameterA |
| 38 |  | Perimeter | N_Perimeter |
| 39 |  | Area | N_Area |
| 40 | Distal Edge of Left Subclavian Artery | Min | B_min |
| 41 |  | Max | B_max |
| 42 |  | Diameter Calculated From Perimeter | B_diameterP |
| 43 |  | Diameter Calculated From Area | B_diameterA |
| 44 |  | Perimeter | B_Perimeter |
| 45 |  | Area | B_Area |
| 46 | Proximal Edge of Celiac Trunk | Min | F_min |
| 47 |  | Max | F_max |
| 48 |  | Diameter Calculated From Perimeter | F_diameterP |
| 49 |  | Diameter Calculated From Area | F_diameterA |
| 50 |  | Perimeter | F_Perimeter |
| 51 |  | Area | F_Area |
| 52 | Proximal Edge of Superior Mesenteric Arery | Min | G_min |
| 53 |  | Max | G_max |
| 54 |  | Diameter Calculated From Perimeter | G_diameterP |
| 55 |  | Diameter Calculated From Area | G_diameterA |
| 56 |  | Perimeter | G_Perimeter |
| 57 |  | Area | G_Area |
| 58 | Proximal Edge of Left Renal Artery | Min | H_min |
| 59 |  | Max | H_max |
| 60 |  | Diameter Calculated From Perimeter | H_diameterP |
| 61 |  | Diameter Calculated From Area | H_diameterA |
| 62 |  | Perimeter | H_Perimeter |
| 63 |  | Area | H_Area |
| 64 | Proximal Edge of Right Renal Artery | Min | I_min |
| 65 |  | Max | I_max |
| 66 |  | Diameter Calculated From Perimeter | I_diameterP |
| 67 |  | Diameter Calculated From Area | I_diameterA |
| 68 |  | Perimeter | I_Perimeter |
| 69 |  | Area | I_Area |
| 70 | Distal Edge of Common Iliac Artery | Min | J_min |
| 71 |  | Max | J_max |
| 72 |  | Diameter Calculated From Perimeter | J_diameterP |
| 73 |  | Diameter Calculated From Area | J_diameterA |
| 74 |  | Perimeter | J_Perimeter |
| 75 |  | Area | J_Area |
| 76 | Distal Edge of Left Iliac Artery | Min | K_min |
| 77 |  | Max | K_max |
| 78 |  | Diameter Calculated From Perimeter | K_diameterP |
| 79 |  | Diameter Calculated From Area | K_diameterA |
| 80 |  | Perimeter | K_Perimeter |
| 81 |  | Area | K_Area |
| 82 | Distal Edge of Right Iliac Artery | Min | L_min |
| 83 |  | Max | L_max |
| 84 |  | Diameter Calculated From Perimeter | L_diameterP |
| 85 |  | Diameter Calculated From Area | L_diameterA |
| 86 |  | Perimeter | L_Perimeter |
| 87 |  | Area | L_Area |

**Supplementary Table 2.** Feature Values in Test and Train sets.

| **Variables** | | **Test** | **Train** | **p** |
| --- | --- | --- | --- | --- |
| Sample Number | | 40 | 160 |  |
| Gender (%) | Female | 16 (40.0) | 57 (35.6) | 0.741 |
|  | Male | 24 (60.0) | 103 (64.4) |  |
| Age (median [IQR]) | | 63.00 [48.75, 71.25] | 60.00 [46.00, 71.00] | 0.555 |
| Height (median [IQR]) | | 165.50 [158.00, 173.25] | 168.00 [160.00, 173.00] | 0.584 |
| Weight (median [IQR]) | | 67.50 [60.00, 74.25] | 65.00 [58.75, 72.50] | 0.635 |
| BMI (median [IQR]) | | 23.99 [22.13, 26.15] | 23.69 [21.18, 25.69] | 0.346 |
| Hypertension (%) | | 18 (45.0) | 71 (44.4) | 1 |
| Blood Presure Control (%) | | 9 (22.5) | 51 (31.9) | 0.335 |
| Dyslipidemia (%) | | 9 (22.5) | 35 (21.9) | 1 |
| Diabetes (%) | | 5 (12.5) | 19 (11.9) | 1 |
| Smoke (%) | | 3 (7.5) | 27 (16.9) | 0.216 |
| Alchol (%) | | 1 (2.5) | 22 (13.8) | 0.086 |
| Autoimmune Disorder (%) | | 2 (5.0) | 12 (7.5) | 0.835 |
| MI (%) | | 0 (0.0) | 7 (4.4) | 0.387 |
| CAD (%) | | 9 (22.5) | 26 (16.2) | 0.485 |
| CKD (%) | | 0 (0.0) | 1 (0.6) | 1 |
| Stroke (%) | | 0 (0.0) | 7 (4.4) | 0.387 |
| A_min (mean (SD)) | | 31.57 (4.41) | 31.10 (5.12) | 0.594 |
| A_max (mean (SD)) | | 34.41 (4.35) | 33.94 (5.27) | 0.607 |
| A_diameterP (mean (SD)) | | 33.16 (4.32) | 32.61 (5.16) | 0.533 |
| A_diameterA (mean (SD)) | | 33.02 (4.32) | 32.48 (5.16) | 0.546 |
| A_Perimeter (mean (SD)) | | 104.20 (13.60) | 102.44 (16.21) | 0.527 |
| A_Area (mean (SD)) | | 870.52 (227.99) | 849.26 (268.20) | 0.645 |
| C_min (mean (SD)) | | 22.98 (4.03) | 23.25 (3.99) | 0.7 |
| C_max (mean (SD)) | | 24.93 (4.54) | 25.09 (4.38) | 0.838 |
| C_diameterP (mean (SD)) | | 24.04 (4.32) | 24.26 (4.18) | 0.76 |
| C_diameterA (mean (SD)) | | 23.96 (4.29) | 24.18 (4.15) | 0.767 |
| C_Perimeter (mean (SD)) | | 75.53 (13.59) | 76.23 (13.13) | 0.765 |
| C_Area (mean (SD)) | | 470.02 (141.63) | 472.64 (166.06) | 0.927 |
| D_min (mean (SD)) | | 21.39 (3.17) | 21.63 (3.47) | 0.69 |
| D_max (mean (SD)) | | 23.21 (3.37) | 23.22 (3.86) | 0.99 |
| D_diameterP (mean (SD)) | | 22.45 (3.26) | 22.51 (3.68) | 0.92 |
| D_diameterA (mean (SD)) | | 22.36 (3.25) | 22.43 (3.64) | 0.911 |
| D_Perimeter (mean (SD)) | | 70.55 (10.26) | 70.72 (11.55) | 0.932 |
| D_Area (mean (SD)) | | 401.05 (119.81) | 405.61 (134.32) | 0.845 |
| E_min (mean (SD)) | | 21.00 (3.31) | 20.70 (3.13) | 0.593 |
| E_max (mean (SD)) | | 22.71 (3.40) | 22.30 (3.46) | 0.506 |
| E_diameterP (mean (SD)) | | 21.93 (3.35) | 21.63 (3.37) | 0.611 |
| E_diameterA (mean (SD)) | | 21.86 (3.33) | 21.52 (3.26) | 0.55 |
| E_Perimeter (mean (SD)) | | 68.90 (10.51) | 67.95 (10.56) | 0.61 |
| E_Area (mean (SD)) | | 383.98 (125.50) | 363.30 (126.76) | 0.356 |
| O_min (mean (SD)) | | 7.59 (1.25) | 7.73 (1.45) | 0.572 |
| O_max (mean (SD)) | | 8.73 (1.42) | 8.70 (1.62) | 0.904 |
| O_diameterP (mean (SD)) | | 8.26 (1.36) | 8.25 (1.50) | 0.971 |
| O_diameterA (mean (SD)) | | 8.17 (1.31) | 8.19 (1.50) | 0.948 |
| O_Perimeter (mean (SD)) | | 25.92 (4.27) | 25.89 (4.73) | 0.963 |
| O_Area (mean (SD)) | | 53.80 (18.22) | 54.44 (19.91) | 0.855 |
| M_min (mean (SD)) | | 7.96 (1.36) | 7.79 (1.26) | 0.437 |
| M_max (mean (SD)) | | 8.88 (1.40) | 8.81 (1.35) | 0.758 |
| M_diameterP (mean (SD)) | | 8.46 (1.36) | 8.35 (1.27) | 0.609 |
| M_diameterA (mean (SD)) | | 8.40 (1.35) | 8.30 (1.26) | 0.634 |
| M_Perimeter (mean (SD)) | | 26.57 (4.26) | 26.22 (3.99) | 0.632 |
| M_Area (mean (SD)) | | 57.01 (19.22) | 55.32 (16.88) | 0.582 |
| N_min (mean (SD)) | | 7.86 (1.30) | 7.81 (1.41) | 0.845 |
| N_max (mean (SD)) | | 8.93 (1.17) | 8.87 (1.45) | 0.808 |
| N_diameterP (mean (SD)) | | 8.46 (1.19) | 8.37 (1.40) | 0.715 |
| N_diameterA (mean (SD)) | | 8.41 (1.20) | 8.33 (1.40) | 0.721 |
| N_Perimeter (mean (SD)) | | 26.58 (3.74) | 26.31 (4.40) | 0.727 |
| N_Area (mean (SD)) | | 56.64 (17.10) | 55.99 (19.64) | 0.849 |
| B_min (mean (SD)) | | 23.74 (3.38) | 24.03 (3.22) | 0.613 |
| B_max (mean (SD)) | | 26.26 (3.98) | 26.79 (3.73) | 0.429 |
| B_diameterP (mean (SD)) | | 25.12 (3.73) | 25.57 (3.44) | 0.469 |
| B_diameterA (mean (SD)) | | 25.01 (3.67) | 25.41 (3.38) | 0.512 |
| B_Perimeter (mean (SD)) | | 78.94 (11.74) | 80.35 (10.79) | 0.468 |
| B_Area (mean (SD)) | | 501.34 (150.43) | 515.84 (137.05) | 0.558 |
| F_min (mean (SD)) | | 19.88 (2.75) | 19.64 (2.84) | 0.633 |
| F_max (mean (SD)) | | 21.98 (2.80) | 21.93 (3.24) | 0.936 |
| F_diameterP (mean (SD)) | | 21.07 (2.73) | 20.93 (3.04) | 0.803 |
| F_diameterA (mean (SD)) | | 20.96 (2.69) | 20.81 (2.98) | 0.763 |
| F_Perimeter (mean (SD)) | | 66.18 (8.55) | 65.77 (9.53) | 0.804 |
| F_Area (mean (SD)) | | 350.70 (90.40) | 347.01 (100.26) | 0.832 |
| G_min (mean (SD)) | | 18.38 (2.65) | 18.45 (2.83) | 0.898 |
| G_max (mean (SD)) | | 20.06 (2.82) | 20.32 (2.97) | 0.623 |
| G_diameterP (mean (SD)) | | 19.27 (2.66) | 19.47 (2.86) | 0.679 |
| G_diameterA (mean (SD)) | | 19.18 (2.66) | 19.39 (2.85) | 0.688 |
| G_Perimeter (mean (SD)) | | 60.51 (8.34) | 61.18 (8.97) | 0.672 |
| G_Area (mean (SD)) | | 294.56 (82.27) | 301.43 (89.15) | 0.658 |
| H_min (mean (SD)) | | 16.71 (2.74) | 16.95 (2.80) | 0.623 |
| H_max (mean (SD)) | | 19.18 (3.03) | 19.24 (2.94) | 0.906 |
| H_diameterP (mean (SD)) | | 18.02 (2.84) | 18.19 (2.83) | 0.727 |
| H_diameterA (mean (SD)) | | 17.89 (2.79) | 18.07 (2.81) | 0.717 |
| H_Perimeter (mean (SD)) | | 56.61 (8.92) | 57.17 (8.89) | 0.724 |
| H_Area (mean (SD)) | | 257.18 (80.83) | 263.86 (79.34) | 0.635 |
| I_min (mean (SD)) | | 16.40 (2.47) | 16.44 (2.85) | 0.943 |
| I_max (mean (SD)) | | 18.45 (2.64) | 18.50 (3.03) | 0.916 |
| I_diameterP (mean (SD)) | | 17.48 (2.49) | 17.56 (2.88) | 0.876 |
| I_diameterA (mean (SD)) | | 17.39 (2.50) | 17.45 (2.86) | 0.903 |
| I_Perimeter (mean (SD)) | | 54.93 (7.86) | 55.16 (9.05) | 0.883 |
| I_Area (mean (SD)) | | 242.22 (70.00) | 246.86 (79.16) | 0.735 |
| J_min (mean (SD)) | | 13.62 (2.03) | 13.67 (2.59) | 0.906 |
| J_max (mean (SD)) | | 15.39 (2.28) | 15.64 (2.63) | 0.591 |
| J_diameterP (mean (SD)) | | 14.58 (2.11) | 14.78 (2.55) | 0.655 |
| J_diameterA (mean (SD)) | | 14.50 (2.10) | 14.67 (2.56) | 0.695 |
| J_Perimeter (mean (SD)) | | 45.80 (6.61) | 46.43 (8.02) | 0.647 |
| J_Area (mean (SD)) | | 168.66 (49.32) | 174.11 (68.46) | 0.637 |
| K_min (mean (SD)) | | 9.48 (1.36) | 9.34 (1.84) | 0.655 |
| K_max (mean (SD)) | | 10.62 (1.45) | 10.59 (2.09) | 0.929 |
| K_diameterP (mean (SD)) | | 10.10 (1.39) | 10.03 (1.96) | 0.832 |
| K_diameterA (mean (SD)) | | 10.05 (1.39) | 9.95 (1.93) | 0.772 |
| K_Perimeter (mean (SD)) | | 31.73 (4.39) | 31.51 (6.16) | 0.828 |
| K_Area (mean (SD)) | | 80.74 (22.59) | 80.75 (33.16) | 0.999 |
| L_min (mean (SD)) | | 9.55 (1.45) | 9.48 (1.74) | 0.809 |
| L_max (mean (SD)) | | 10.67 (1.65) | 10.82 (2.36) | 0.703 |
| L_diameterP (mean (SD)) | | 10.18 (1.52) | 10.21 (2.04) | 0.931 |
| L_diameterA (mean (SD)) | | 10.13 (1.52) | 10.12 (1.92) | 0.986 |
| L_Perimeter (mean (SD)) | | 31.98 (4.79) | 32.07 (6.41) | 0.938 |
| L_Area (mean (SD)) | | 82.29 (25.21) | 83.34 (33.96) | 0.856 |

**Supplementary Table 3.** Basic characteristics of three TEVAR patients.

| **Patient Num** | **Patient 1** | **Patient 2** | **Patient 3** | **Patient 4** | **Patient 5** |
| --- | --- | --- | --- | --- | --- |
| Gender | Male | Male | Male | Female | Male |
| Age | 72 | 64 | 64 | 65 | 64 |
| Height (cm) | 168 | 172 | 170 | 160 | 172 |
| Weight (kg) | 68.9 | 90 | 67 | 60 | 68 |
| BMI (kg/m^2^) | 24.41 | 30.42 | 23.18 | 23.44 | 22.99 |
| Hypertension | Yes | Yes | No | Yes | Yes |
| Blood Pressure Control | No | Yes | No | Yes | Yes |
| Dyslipidemia | No | No | No | No | No |
| Diabetes | No | No | No | No | No |
| Smoke | No | No | No | No | No |
| Alchol | No | No | No | No | No |
| Autoimmune Disorder | No | No | No | No | No |
| Myocardiac Infarction | No | Yes | No | No | No |
| CAD | No | Yes | No | No | No |
| CKD | No | No | No | No | No |
| Stroke | Yes | No | No | Yes | No |

## Supplementary Figures


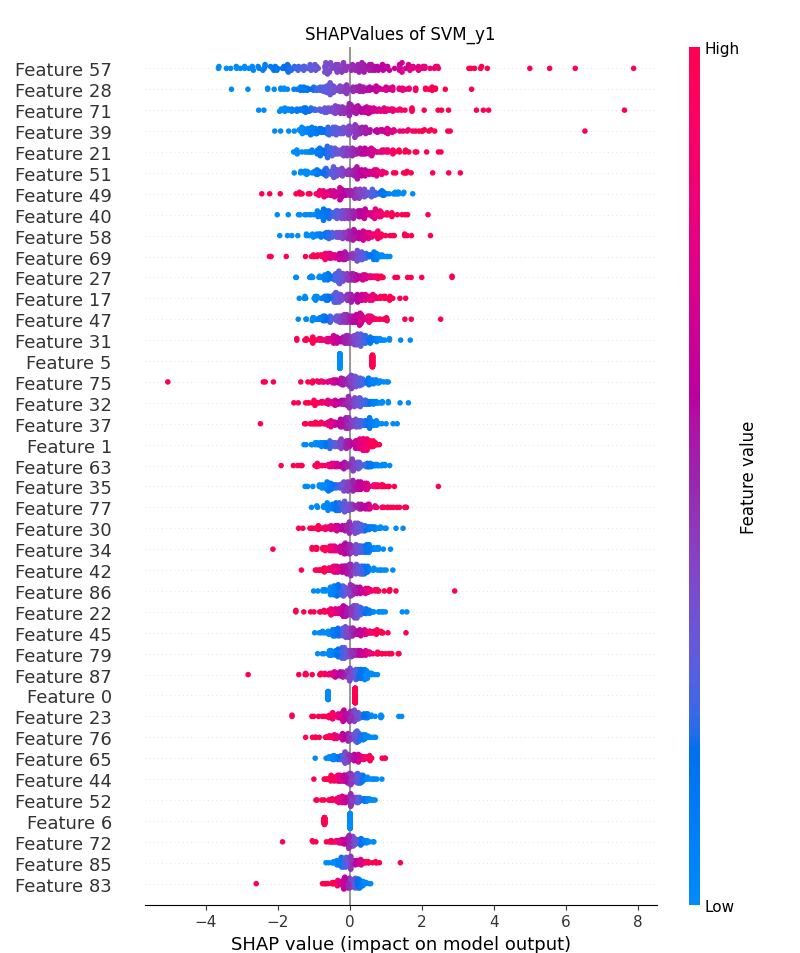


**Supplementary Figure Yes.** Summary plot of SVM models containing 40 features at y_Yes_.


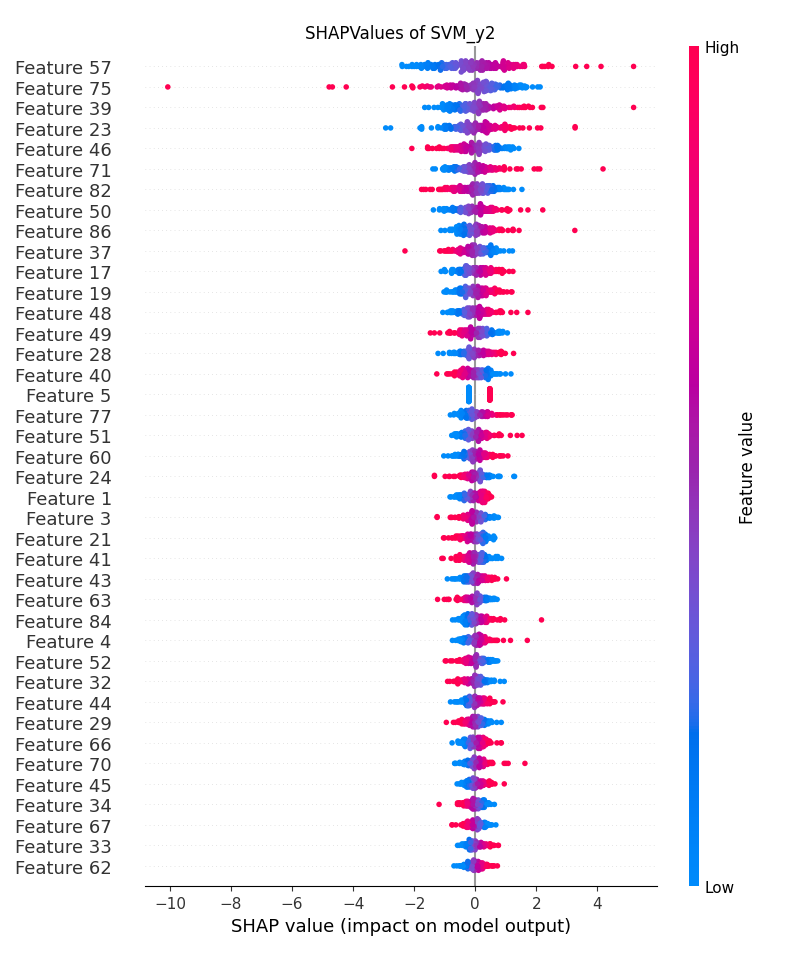


**Supplementary Figure 2.** Summary plot of SVM models containing 40 features at y_2_.


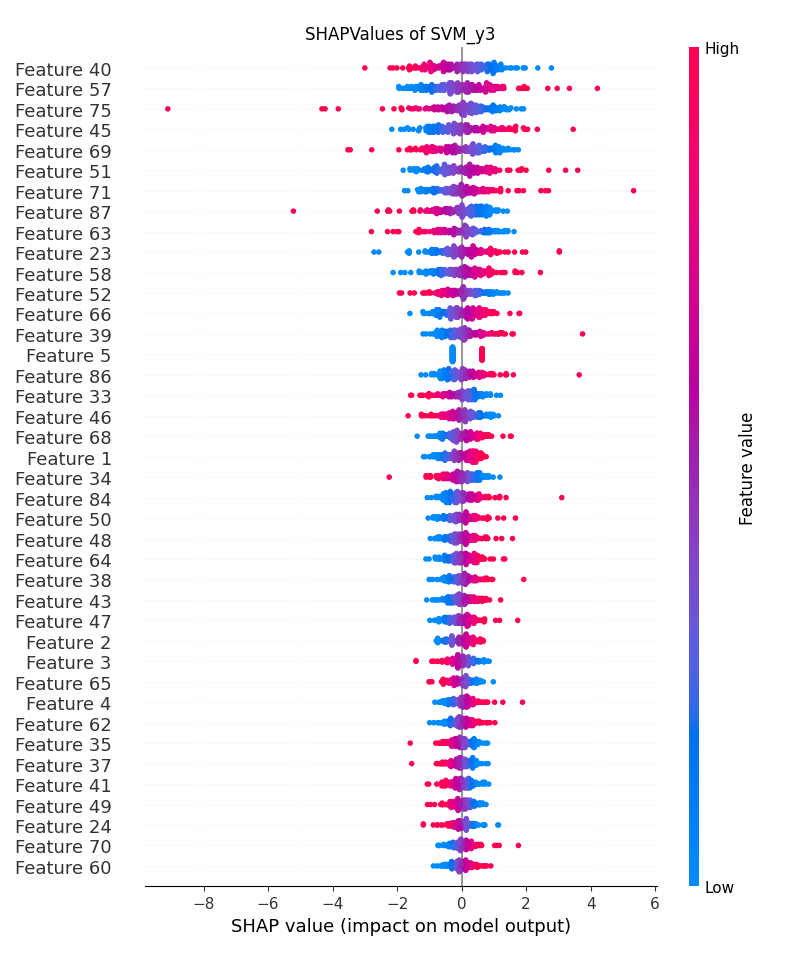


**Supplementary Figure 3.** Summary plot of SVM models containing 40 features at y_3_.
